# Are the American Society for Radiation Oncology Guidelines Accurate Predictors of Recurrence in Early Stage Breast Cancer Patients Treated with Balloon-Based Brachytherapy?

**DOI:** 10.1155/2013/829050

**Published:** 2013-12-08

**Authors:** Moira K. Christoudias, Abigail E. Collett, Tari S. Stull, Edward J. Gracely, Thomas G. Frazier, Andrea V. Barrio

**Affiliations:** ^1^Department of Surgery, The Bryn Mawr Hospital, Bryn Mawr, PA 19010, USA; ^2^Drexel University College of Medicine, Drexel University, School of Public Health, Philadelphia, PA 19129, USA

## Abstract

The American Society for Radiation Oncology (ASTRO) consensus statement (CS) provides guidelines for patient selection for accelerated partial breast irradiation (APBI) following breast conserving surgery. The purpose of this study was to evaluate recurrence rates based on ASTRO CS groupings. A single institution review of 238 early stage breast cancer patients treated with balloon-based APBI via balloon based brachytherapy demonstrated a 4-year actuarial ipsilateral breast tumor recurrence (IBTR) rate of 5.1%. There were no significant differences in the 4-year actuarial IBTR rates between the “suitable,” “cautionary,” and “unsuitable” ASTRO categories (0%, 7.2%, and 4.3%, resp., *P* = 0.28). ER negative tumors had higher rates of IBTR than ER positive tumors. The ASTRO groupings are poor predictors of patient outcomes. Further studies evaluating individual clinicopathologic features are needed to determine the safety of APBI in higher risk patients.

## 1. Introduction

The concept of treating the entire breast with radiation after breast conserving surgery for breast cancer arose from early data demonstrating a high incidence of invasive or in situ carcinoma remote from the primary tumor in mastectomy specimens [1, 2]. However, clinical data evaluating patterns of recurrence have demonstrated that patients treated with lumpectomy alone have a low rate of recurrences at remote sites, so-called “elsewhere” recurrences. In fact, less than 5% of recurrences in unirradiated patients occur in a quadrant away from the primary tumor and the addition of whole breast irradiation (WBI) has not been shown to reduce the rates of these “elsewhere” failures [3]. Based on documented patterns of breast tumor recurrence after breast conserving surgery, accelerated partial breast irradiation (APBI) emerged as an alternative to WBI [4, 5]. However, data from randomized controlled trials comparing the safety and efficacy of APBI with standard WBI will not be available for several years [6].

In response to the growing interest in APBI, the American Society for Radiation Oncology (ASTRO) Health Services Research Committee convened a Task Force to help guide patient selection for APBI use outside of a clinical trial. In July 2009, the Task Force published a consensus statement (CS) that classified patients as “suitable,” “cautionary,” and “unsuitable” based on patient and tumor characteristics [7]. It is noteworthy that CS groupings were constructed largely without the use of long-term clinical data on the efficacy of APBI and may not be ideal in identifying appropriate patients for this modality [7].

Few large series have evaluated patient outcomes with APBI based on ASTRO CS guidelines and less so with APBI using solely balloon-based brachytherapy techniques [8–10]. This study reviews our single institution-experience with balloon-based APBI in patients with early stage breast cancer and evaluates recurrence rates based on ASTRO CS groupings.

## 2. Materials and Methods

Following approval by our institutional review board, we performed a retrospective chart review of patients treated with APBI at our institution between March 2004 and May 2010. We identified 238 patients with 243 early stage breast cancers of which 2 had bilateral breast cancer and 3 had metachronous contralateral breast cancers. All patients underwent breast conserving surgery performed by one of two surgeons followed by APBI via balloon catheter brachytherapy. Patients were classified as “suitable,” “cautionary,” or “unsuitable” based on ASTRO CS guidelines (Table 1).

All clinicopathologic factors were used to assign consensus panel groups, except for BRCA status as information regarding genetic testing was not readily available on most patients. A patient was considered estrogen receptor (ER) positive if the breast tumor contained at least 1% ER positive cells [11]. ER status was unknown for one patient due to a technical error during immunohistochemical staining. She was placed into the “cautionary” group based on tumor histology (invasive lobular carcinoma) and was included in the analysis. Data regarding lymphovascular invasion (LVI) was available for all patients but assessed as indeterminate by the pathologist in 17 of the 243 cancers (7%). These patients were placed into their respective ASTRO categories based on their other clinicopathologic features. Patients with any one “cautionary” or “unsuitable” criterion were placed in that respective category.

Two hundred nine cancers (86.0%) were treated with MammoSite single lumen catheters, 12 (4.9%) with MammoSite multilumen catheters, and 1 (0.4%) with a MammoSite ellipsoidal catheter (Hologic Inc., Bedford, MA). Twenty-one cancers (8.6%) were treated with Contura multilumen catheters (SenoRx Inc., Irvine, CA).

### 2.1. Catheter Insertion Technique

One hundred thirty of the 243 balloons (53.5%) were placed percutaneously in the office via a lateral incision or an incision at inframammary fold. One hundred thirteen (46.5%) balloons were placed in the operating room, 61 (54.0%) were placed via an open technique, and 52 (46.0%) were placed percutaneously. All of the balloons placed in the operating room were placed at a separate operation from the initial lumpectomy. All patients had preplacement ultrasound confirming adequate skin-to-seroma distance ≥7 mm.

### 2.2. Radiation Treatment

Our accelerated radiation treatment technique has been previously described in detail [12]. Each patient was seen by radiation oncology for computed tomography-based 3D treatment planning within 48 hours of balloon placement. Patients received 34 Gy delivered 1 cm from the balloon surface, in two fractions of 3.4 Gy each per day, 6 hours apart over 5 treatment days.

### 2.3. Outcome Measure

Ipsilateral breast tumor recurrence (IBTR) was defined as recurrence of the cancer in the treated breast. Each recurrence was classified by the investigator as either a true recurrence (occurring within 2 cm of the lumpectomy bed) or an elsewhere recurrence, based on the criteria by Recht et al. [13].

### 2.4. Statistical Analysis

Kaplan-Meier curves were used to generate time-to-event curves for IBTR. Statistical significance of differences between groups was calculated using log rank tests (for Kaplan-Meier curves). Predictions of IBTR over time for individual clinical and pathologic factors were obtained with Cox proportional Hazards Models, which provided a hazard ratio and 95% confidence interval. When one of the groups had no events (such as LVI), the Cox model could not be used and a comparison with the Fisher exact test was instead used to obtain a *P *value.

## 3. Results

Of the 243 breast cancers, 58 (24%) were classified as “suitable,” 119 (49%) as “cautionary,” and 66 (27%) as “unsuitable” per the ASTRO guidelines.  Table 2 describes the breakdown of the study cohort according to CS groupings. Lymph node status and extensive intraductal component (EIC) are reported only for patients with invasive carcinoma (*n* = 72 “cautionary,” *n* = 50 “unsuitable”). The 119 cancers in the “cautionary” group met 182 “cautionary” criteria, with 50 (42%) meeting more than one “cautionary” criterion. The majority of “cautionary” patients had either close margins (27%) or DCIS ≤ 3 cm (27%). ER negative tumors comprised 31 (26%) of the “cautionary” cases (invasive, *n* = 22; DCIS, *n* = 9) and 10 (15%) of the “unsuitable” cases (invasive, *n* = 6; DCIS, *n* = 4). Of the 66 cancers in the “unsuitable” group, 11 (17%) met more than one “unsuitable” criterion for a total of 77 criteria. Patients under the age of 50 or with positive margins comprised 73% of the “unsuitable” cases.

### 3.1. Ipsilateral Breast Tumor Recurrences

**Figure 1:**
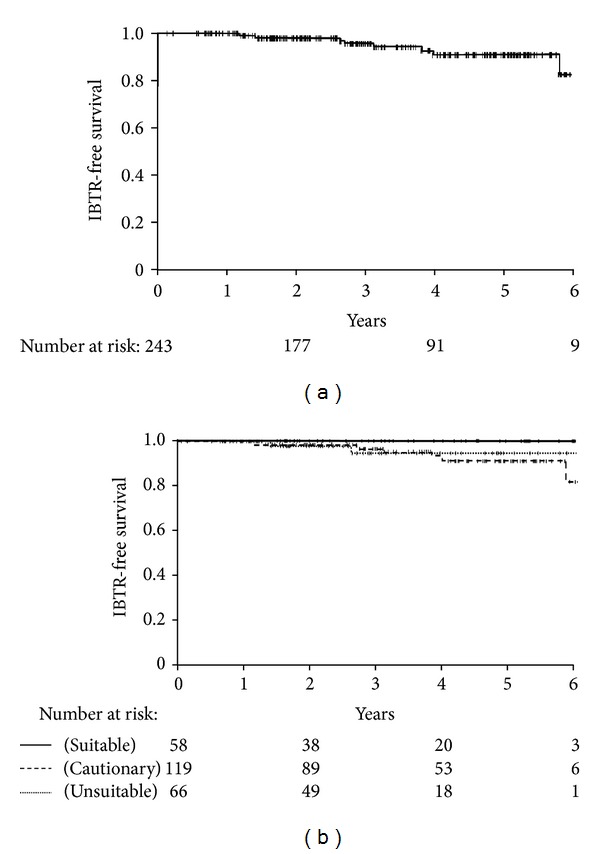
(a) Ipsilateral breast tumor recurrence-free survival for 243 breast cancers. (b) Ipsilateral breast tumor recurrence-free survival stratified by ASTRO consensus statement grouping.

The median followup was 3.2 (range, 0.2–7.1) years. There were 8 IBTR (3.2%) at a median of 2.9 years, of which one was a true local recurrence and 7 were elsewhere recurrences. The 4-year actuarial IBTR rate for the entire cohort was 5.1% (Figure 1(a)). There were no significant differences in the 4-year actuarial IBTR rates between the “suitable,” “cautionary,” and “unsuitable” categories (0%, 7.2%, and 4.3% resp., *P* = 0.28) (Figure 1(b)). Three (4.8%) of the 63 patients with DCIS developed an IBTR (1 local, 2 elsewhere). The 4-year rate of IBTR was not different between patients with DCIS and invasive carcinoma (4.3% versus 5.4%, *P* = 0.51). There were 3 IBTR among 28 patients with ER negative invasive cancers versus 2 of 151 ER positive (crude rate 10.7% versus 1.3%, resp.,). Patients with ER negative invasive cancers had a significantly higher 4-year actuarial IBTR rate compared with ER positive patients (16.5% versus 1.9%, *P* = 0.03).

Of the 8 IBTR, 3 (37.5%) occurred in patients with ER negative invasive tumors (2 triple negative, 1 ER negative/HER-2/neu positive). All 3 ER negative recurrences were elsewhere recurrences, which is similar to the percentage of elsewhere recurrences (4 of 5) in the remainder of the cohort (100% versus 80%, *P* = 0.6). On univariate analysis, patients with ER negative invasive tumors had a higher risk of IBTR, although this did not reach statistical significance (HR = 5.87, *P* = 0.053). Patients with close/positive margins appeared to be associated with a higher risk of IBTR (HR 7.63, *P* = 0.02); however only one of the IBTR was a true local recurrence which occurred in a young patient (age 50−59) with DCIS ≤3 cm and negative surgical margins at initial surgery. Age, tumor size, histology, multifocality, LVI, and nodal status were not associated with IBTR (Table 3).

Table 4 shows the tumor characteristics and surgical management of the patients experiencing an IBTR. Five (62.5%) of the 8 IBTR were treated with repeat breast conservation with 2 patients agreeing to additional radiation therapy (1 WBI, 1 APBI). Three of the 8 IBTR, or 1.2% of the entire cohort, were treated with salvage mastectomy. After their in breast recurrences, four patients underwent genetic testing at the discretion of the treating physician. One was found to carry a deleterious mutation in the BRCA1 gene.

### 3.2. Regional and Distant Recurrences

There were 5 (2.1%) regional recurrences, all axillary, at a median disease free interval of 2.2 years. One was initially classified as “cautionary” and 4 were “unsuitable.” Two had concomitant distant metastases at the time of regional recurrence. There were two additional isolated distant metastases. Of the 4 distant metastases, all were originally classified as “unsuitable” and occurred at a median of 1.9 years. Among the 28 ER negative patients, the crude rate of regional and distant recurrence was 7.1% and 3.6%, respectively.

## 4. Discussion

Data from a recently published Medicare claims database demonstrated an increase in APBI use in women diagnosed with invasive carcinoma from 3.47% in 2003 to 12.52% in 2007 [14]. As a result of the growing popularity of APBI, ASTRO published a CS to provide guidance to physicians and patients regarding the use of APBI outside of a clinical trial. Based on review of the literature and expert opinion, patients considered for APBI were stratified into three major groups: “suitable,” “cautionary,” and “unsuitable” [7]. It is important to note that the CS groupings were not based on data that identified subsets of patients with higher or lower rates of in breast recurrence with APBI but rather a lack of data supporting its use. Although the ASTRO CS guidelines have been applied to MammoSite Registry data, as well as other single institution series, additional studies are needed to aid in continued refinement of the ASTRO CS groupings [8, 10]. We applied the ASTRO CS groupings to 243 early stage cancers treated with balloon-based brachytherapy at our institution to evaluate recurrence patterns by group. In addition, we evaluated individual clinicopathologic features to identify factors predictive of IBTR in patients treated with balloon-based brachytherapy.

We observed a 4-year actuarial IBTR rate of 5.1% for the entire cohort. Our 4-year recurrence rate is slightly higher than the 3.9% 5-year actuarial rate observed in the MammoSite Registry trial which may be related to differences in patient and tumor characteristics in each study [15]. When stratified by ASTRO CS groupings, there was no significant difference between the “suitable,” “cautionary,” and “unsuitable” patient cohorts (0%, 7.2%, and 4.3%, resp., *P* = 0.28). Our data is consistent with Shaitelman et al. [10] who demonstrated no difference in 5-year IBTR rates for all CS groupings in their 1,025 patients from the MammoSite Registry trial (2.59%, 5.43%, and 5.28%, resp., *P* = 0.19). In a recent study, Vicini et al. [8] likewise reported no difference in 10-year rates of IBTR based on ASTRO CS groups (*P* = 0.86). Similar to our study, Vicini et al. [8] did report a slightly higher (nonsignificant) IBTR rate (7.8%) in “cautionary” patients receiving APBI compared to the other groupings. It is likely that ER negative invasive cancers, as demonstrated by a higher 4-year actuarial IBTR rate of 16.5%, may be driving the observed, albeit nonsignificant higher rate of IBTR among “cautionary” patients in our dataset. Initially, there appeared to be an association between close/positive margins and IBTR. However, since only one of the 8 IBTR was a true recurrence, this finding likely does not reflect a true association between margins and IBTR. Other “cautionary” and “unsuitable” characteristics such as age, tumor size, histology, multifocality, LVI, and nodal status did not seem to affect IBTR. Based on accumulating data, the ASTRO CS groupings as a whole, are poor predictors of patient outcomes. Appropriateness for APBI should be based on individual clinical and pathologic features of the tumor.

Patients with ER negative invasive tumors treated with APBI have been shown to have a higher risk of IBTR in several studies. Data from the MammoSite Registry Trial demonstrated that negative ER status was the only variable, associated with IBTR in patients with invasive carcinoma (OR 4.01, *P* = 0.003) [10]. Similarly, Wilder et al noted that ER negative tumors treated with APBI had inferior locoregional control, albeit nonsignificant (*P* = 0.09) and a worse relapse-free survival (*P* = 0.04) [16]. In our study cohort, patients with ER negative invasive cancers also had a higher rate of IBTR (HR = 5.87), although this did not reach statistical significance (*P* = 0.053). Given the small total number of ER negative invasive cancers (*n* = 28), as well as the low absolute number of recurrences in our study, we may be underpowered to demonstrate an association, even if one exists.

Further analysis of our data demonstrated a crude rate of IBTR of 10.7% in our ER negative cohort and 4-year actuarial IBTR rate of 16.5%. Although our crude rate of IBTR appears similar to the 10.1% crude IBTR rate reported by Beitsch et al. [17] in their 139 ER negative MammoSite Registry patients, our rate is higher when taking into account median follow-up time (3.2 years versus 5 years, resp.,). Moreover, the 16.5% 4-year rate of IBTR is higher than the 8-year IBTR rate of 13.4% seen in ER negative patients treated with WBI in the absence of adjuvant chemotherapy in the National Surgical Adjuvant Bowel and Breast project (NSABP) B-13 study [18]. The higher IBTR rate among ER negative patients in our study may be explained by the finding of a BRCA mutation in one of the 3 ER negative patients that recurred. Due to the small number of ER negative patients as well as the small number of absolute recurrences, any one relapse will dramatically affect the overall recurrence rate. Excluding this patient would result in a lower crude IBTR rate (7.4%), which is more consistent with the MammoSite Registry data [17]. The recurrence rate is still higher than that of ER positive patients in this study (crude IBTR rate 1.3%) and raises concerns regarding the safety of APBI in this group of patients. Whether these patients would have had a lower IBTR rate with WBI is unknown. Poor tumor biology, rather than inferiority of APBI, may be driving the increased IBTR rate in ER negative patients seen in this and other studies. Until mature data from randomized clinical trials comparing APBI and WBI become available, patients with ER negative invasive tumors should be treated with APBI inside of a clinical trial.

Alternatively, patients with ER positive invasive cancers (*n* = 151) had a 1.9% 4-year actuarial IBTR rate. The low IBTR rate is consistent with the 1.1% rate of local recurrence seen in ER positive patients treated with WBI in NSABP B-14 at 4 years of followup [19]. Our data support the evidence that patients with ER positive invasive cancers can be safely treated with accelerated partial breast irradiation, with excellent local control.

Our study has several limitations. It is a nonrandomized retrospective review of a modest cohort of patients. Furthermore, the number of patients meeting individual ASTRO criteria is unbalanced because selection for APBI in these patients was at the discretion of the treating physician. Therefore, there are more patients fulfilling certain criteria (ER positive, DCIS, and close margins) and fewer meeting other criteria (ER negative, LVI, and EIC). This, along with a small absolute number of recurrences, may make our study underpowered to demonstrate an association between certain clinical and pathologic factors and risk of IBTR. Following their recurrences, one patient was discovered to carry a deleterious mutation in the BRCA 1 gene. As this patient was not excluded, this may bias our results, resulting in a higher than expected IBTR rate, particularly among ER negative invasive cancers. Finally, our median followup is short. Longer followup is needed to ensure that patients treated with APBI have an acceptably low rate of IBTR.

In conclusion, an analysis of 243 breast cancers treated with balloon-based brachytherapy demonstrated no significant difference in recurrence rates based on ASTRO CS groupings. Patients with ER positive invasive cancers demonstrated low rates of IBTR and can be safely treated with APBI with excellent local control. Conversely, patients with ER negative invasive tumors had a higher rate of IBTR compared with historical controls treated with WBI, which resulted in a higher than expected IBTR for the entire cohort. Although poor tumor biology, rather than inferior disease control with APBI, may be responsible for the worse outcomes, strong consideration should be given in treating patients with ER negative invasive cancers with APBI inside of a clinical trial. Other individual ASTRO criteria were not associated with an increased risk of IBTR. The ASTRO CS groupings are poor predictors of suitability for APBI and decision for treatment should be based on individual clinical and pathologic features.

## Figures and Tables

**Table 1:** American Society for Radiation Oncology (ASTRO) guidelines.

Characteristic	ASTRO CS grouping		
	Suitable	Cautionary	Unsuitable
Age, y	≥60	50–59	<50
Tumor size, cm	≤2	2.1–3.0	>3
T stage	T1	T0 or T2	T3-T4
Histology	Invasive ductal or other favorable subtypes	Invasive lobular or DCIS ≤ 3 cm	DCIS > 3 cm
ER status	Positive	Negative	NA
Grade	Any	NA	NA
Margins	Negative (≥2 mm)	Close (<2 mm)	Positive
Multifocality, cm	≤2	2.1–3	>3
Multicentricity	Unicentric	NA	Present
LVI	None	Limited/focal	Extensive
EIC	None	Yes and tumor size ≤ 3 cm	Yes and tumor size > 3 cm
Nodal status	Negative	NA	Positive
Nodal surgery	SLNB or ALND	NA	None performed
BRCA 1/2 mutation	Not present	NA	Present
Neoadjuvant therapy	Not allowed	NA	Used

CS: consensus statement, DCIS: ductal carcinoma in situ, ER: estrogen receptor, LVI: lymphovascular invasion, EIC: extensive intraductal component, SLNB: sentinel lymph node biopsy, and ALND: axillary lymph node dissection.

**Table 2:** Breakdown of patient cohort by individual clinical and pathologic characteristics stratified by ASTRO CS grouping.

Characteristic	ASTRO CS grouping No. of patients (%)		
	Suitable *N* = 58	Cautionary *N* = 119	Unsuitable *N* = 66
Age, yrs			
≥60	58 (100)	89 (75)	34 (52)
50–59	0 (0)	30 (25)	4 (6)
<50	0 (0)	0 (0)	28 (42)
Unknown^a^	0 (0)	0 (0)	0 (0)
Tumor Size, cm			
≤2	58 (100)	63 (53)	40 (61)
2.1–3.0	0 (0)	9 (8)	7 (11)
>3	0 (0)	0 (0)	3 (4)
DCIS ≤ 3	0 (0)	47 (39)	14 (21)
DCIS > 3	0 (0)	0 (0)	2 (3)
Unknown^a^	0 (0)	0 (0)	0 (0)
Histology			
IDC	56 (97)	60 (51)	45 (68)
ILC	0 (0)	9 (8)	5 (8)
DCIS	0 (0)	47 (39)	16 (24)
IDC/ILC	2 (3)	2 (1)	0 (0)
Adenoid cystic	0 (0)	1 (1)	0 (0)
Unknown^a^	0 (0)	0 (0)	0 (0)
ER status			
Positive	58 (100)	87 (73)	56 (85)
Negative	0 (0)	31 (26)	10 (15)
Unknown^a^	0 (0)	1 (1)	0 (0)
Margins			
Negative, ≥2 mm	58 (100)	72 (61)	21 (32)
Close, <2 mm	0 (0)	47 (39)	17 (26)
Positive	0 (0)	0 (0)	28 (42)
Unknown^a^	0 (0)	0 (0)	0 (0)
Multifocality			
None	58 (100)	115 (97)	61 (92)
≤2 cm	0 (0)	4 (3)	3 (5)
2.1 cm–3.0 cm	0 (0)	0 (0)	1 (1.5)
>3 cm	0 (0)	0 (0)	1 (1.5)
Unknown^a^	0 (0)	0 (0)	0 (0)
Multicentricity			
Unicentric	58 (100)	119 (100)	66 (100)
Multicentric	0 (0)	0 (0)	0 (0)
Unknown^a^	0 (0)	0 (0)	0 (0)
LVI			
None	54 (93)	107 (90)	52 (79)
Limited/focal	0 (0)	7 (6)	3 (4.5)
Extensive	0 (0)	0 (0)	3 (4.5)
Indeterminate	4 (7)	5 (4)	8 (12)
Unknown	0 (0)	0 (0)	0 (0)
EIC, cm^b^			
None	58 (100)	70 (97)	47 (94)
≤3	0 (0)	2 (3)	2 (4)
>3	0 (0)	0 (0)	1 (2)
Unknown^a^	0 (0)	0 (0)	0 (0)
Nodal status^b^			
Negative	58 (100)	72 (100)	39 (78)
Positive	0 (0)	0 (0)	7 (14)
None performed	0 (0)	0 (0)	4 (8)
Unknown^a^	0 (0)	0 (0)	0 (0)

CS: consensus statement, DCIS: ductal carcinoma in situ, IDC: invasive ductal carcinoma, ILC: invasive lobular carcinoma, ER: estrogen receptor, LVI: lymphovascular invasion, and EIC: extensive intraductal component.

^a^Patients with tumor characteristics characterized as unknown or indeterminate were placed in ASTRO categories based on the other criteria.

^b^Excluding DCIS; cautionary *N* = 72; unsuitable *N* = 50.

**Table 3:** Factors associated with ipsilateral breast tumor recurrence.

Variable	Hazard ratio (95% CI)	*P*
Age: ≥50 versus <50	0.63 (0.08–5.19)	0.67
Age: ≥60 versus <60	0.55 (0.13–2.32)	0.42
Tumor size: >2 cm versus ≤2 cm^a^	2.20 (0.25–19.75)	0.48
Tumor type: ILC versus other	1.89 (0.23–15.41)	0.55
Tumor type: DCIS versus invasive cancer	1.61 (0.38–6.75)	0.52
ER status: negative versus positive^a^	5.87 (0.97–35.34)	0.053
Margins: negative versus close/positive	7.63 (1.45–40.0)	0.016
Multifocality: yes versus no	2.36 (0.28–19.99)	0.43
LVI: present versus none^a,b^	—	0.60
Nodal status: positive versus negative^a,b^	—	1.0

CI: confidence interval, ILC: invasive lobular carcinoma, DCIS: ductal carcinoma in situ, ER: estrogen receptor, and LVI: lymphovascular invasion.

^a^Excludes DCIS.

^b^Cox proportional hazards model could not be performed due to lack of events.

**Table 4:** Surgical management of ipsilateral breast tumor recurrences.

Case no.	Date of surgery	Time to IBTR (yrs)	Location	ASTRO criteria	Surgical management	Radiation therapy	Follow-up time (yrs)	Disease status
22	2005	3.99	Elsewhere	ER negative, close margin	Mastectomy	No	5.21	NED
44	2005	3.13	Elsewhere	ER negative	Mastectomy	No	5.33	NED
50	2005	5.83	Local	Age 50–59, DCIS ≤ 3 cm, and ER negative	Segmental resection	No^a^	5.81	NED
71	2006	2.64	Elsewhere	Age < 50, close margin, and DCIS ≤ 3 cm	Mastectomy	No	5.16	DOD
91.1^b^	2007	3.82	Elsewhere	ER negative, close margin, and tumor size 2.1–3.0 cm	Segmental resection	No^c^	3.95	NED
107	2006	1.45	Elsewhere	Positive margin, multifocal 2.1–3.0 cm	Segmental resection	Yes	4.52	NED
147	2007	2.73	Elsewhere	Close margin	Segmental resection	Yes^d^	3.50	NED
208	2009	1.21	Elsewhere	Close margin, age 50–59, and DCIS ≤ 3 cm	Segmental resection	No^c^	1.56	NED

ER: estrogen receptor, NED: no evidence of disease, DCIS: ductal carcinoma in situ, and DOD: dead of disease.

^a^Patient is currently receiving chemotherapy. Decision for radiation was undetermined at the completion of the study.

^b^Patient tested positive for deleterious mutation in BRCA gene following recurrence.

^c^Patient declined radiation therapy.

^d^Recurrence treated with APBI via balloon catheter brachytherapy.
